# Towards developing a Core Outcome Set for malnutrition intervention studies in older adults: a scoping review to identify frequently used research outcomes

**DOI:** 10.1007/s41999-022-00617-5

**Published:** 2022-03-12

**Authors:** M. Visser, N. Mendonça, C. Avgerinou, T. Cederholm, A. J. Cruz-Jentoft, S. Goisser, E. Kiesswetter, H. M. Siebentritt, D. Volkert, G. Torbahn

**Affiliations:** 1 Department of Health Sciences, Faculty of Science, Vrije Universiteit Amsterdam, Amsterdam Public Health Research Institute, De Boelelaan 1085, 1081 HV Amsterdam, The Netherlands; 2 EpiDoC Unit, CEDOC, NOVA Medical School, Universidade Nova de Lisboa, Lisbon, Portugal; 3 Comprehensive Health Research Centre (CHRC), NOVA Medical School, Universidade Nova de Lisboa, Lisbon, Portugal; 4 Department of Primary Care and Population Health, UCL Research, London, UK; 5 Department of Public Health and Caring Sciences, Clinical Nutrition and Metabolism, Uppsala University, Uppsala, Sweden; 6 Servicio de Geriatría, Hospital Universitario Ramón y Cajal (IRYCIS), Madrid, Spain; 7 Center for Geriatric Medicine, AGAPLESION Bethanien Hospital Heidelberg, Heidelberg University Hospital, Heidelberg, Germany; 8 Institute for Biomedicine of Aging, Friedrich-Alexander-Universität Erlangen-Nürnberg, Kobergerstr. 60, 90408 Nürnberg, Germany; 9 Department of Pediatrics, Paracelsus Medical University, Nuernberg, Germany

**Keywords:** Undernutrition, Aging, ONS, Dietary counselling

## Abstract

**Purpose:**

To conduct a scoping review to provide a systematic overview of outcomes used in nutritional intervention studies focused on the treatment of protein-energy malnutrition in older adults.

**Methods:**

A systematic search of four electronic databases (Medline, EMBASE, CINAHL and Cochrane Central Register of Controlled Trials (CENTRAL) was performed to retrieve randomized controlled trials (RCTs), published until March 9, 2020, that evaluated the effect of nutritional interventions to treat protein-energy malnutrition in older adults and those at risk for malnutrition. Two authors screened titles, abstracts and full texts independently. One author extracted data that were cross-checked by another author.

**Results:**

Sixty-three articles reporting 60 RCTs were identified. Most frequently used outcomes included body weight/body mass index (75.0% of RCTs), dietary intake (61.7%), functional limitations (48.3%), handgrip strength (46.7%), and body circumference (40.0%). The frequencies differed by setting (community, hospital and long-term care). For some outcomes there was a preferred assessment method (e.g., Barthel index for functional limitations), while for other outcomes (e.g., functional performance) a much greater variation was observed.

**Conclusion:**

A large variation in outcomes, not only across but also within settings, was identified in nutritional intervention studies in malnourished older adults and those at risk. Furthermore, for many outcomes there was a large variation in the used assessment method. These results highlight the need for developing a Core Outcome Set for malnutrition intervention studies in older adults to facilitate future meta-analyses that may enhance our understanding on the effectiveness of treatment.

**Supplementary Information:**

The online version contains supplementary material available at 10.1007/s41999-022-00617-5.

## Key Summary Points

**Aim:**

As a first step in developing a Core Outcome Set, we performed a scoping review using a systematic methodology to identify used outcomes in nutritional intervention studies in malnourished older adults and those at risk.

**Findings:**

A large variation in used outcomes and primary outcomes was identified, with considerable differences in the frequency of outcomes between settings. For most outcomes no preferred assessment method could be recognised.

**Message:**

A large heterogeneity in used outcomes and methods to assess outcomes was observed, highlighting the need to develop a Core Outcome Set in order to facilitate future evidence syntheses (e.g. meta-analyses).

**Supplementary Information:**

The online version contains supplementary material available at 10.1007/s41999-022-00617-5.

## Introduction

Despite increasing scientific interest in the topic of malnutrition in older persons over the last decades, many uncertainties remain regarding the effectiveness of nutritional interventions [1–4]. Individual randomized controlled trials (RCTs) show mixed findings, which could be caused by differences in sample size, selection of subjects, type and duration of the intervention, setting, selected outcome(s) and assessment, and overall quality of the conducted research.

The heterogeneity in treatment effects can be investigated using meta-analyses and its cause explored by performing subgroup analyses. Individual participant data (IPD) meta-analyses specifically allow the investigation of subject-level and study-level sources of this heterogeneity in treatment effects [5]. In IPD analyses potential interactions between treatment and factors such as study setting, type of intervention or participants’ characteristics can be thoroughly investigated. Thus, IPD meta-analyses are helpful to increase our understanding on the effectiveness of malnutrition interventions in older adults and to identify who will benefit most from which treatment and in what setting.

A previous IPD meta-analysis investigating the effectiveness of malnutrition intervention in older adults was hampered by several factors [6], including limited availability of individual-based datasets of previously conducted trials (e.g., data were destroyed or could not be shared), limited number of variables regarding subject characteristics (e.g., no information on malnutrition status or dietary intake at baseline), and a large variation in used outcome measures between trials. Thus, only a limited number of trials could be included in the pooled analysis for a specific outcome.

To overcome the latter problem and to support future meta-analyses, the idea to develop a Core Outcome Set (COS) for malnutrition intervention studies in older adults was raised within the Joint Action Malnutrition in the Elderly Knowledge Hub (MaNuEL) [7]. A COS is an agreed minimum set of outcomes that should be measured and reported in all clinical trials of a specific disease or trial population [8]. The development of a COS for malnutrition intervention studies in older adults serves two main purposes: first, stimulate the inclusion of relevant outcome variables to test the effectiveness of a nutritional intervention in malnourished older adults or those at risk; second, decrease the heterogeneity between malnutrition intervention studies in older adults that will benefit future (IPD) meta-analyses.

No COS for malnutrition intervention studies in older adults is yet available. However, three proposals for a Minimum Data Sets (MDS) for intervention studies in older adults were identified: the Geriatric Minimum Data Set [9], the Minimum Data Set 3.0 Resident Assessment Instrument [10], and a MDS for nutritional intervention studies in older adults [11]. Unfortunately, these previously published MDSs cannot serve as a basis for a COS. The main reasons include: not targeting malnourished older persons or those at risk; not targeting nutritional interventions; limited to one setting only; no focus on outcome variables; or the measurement instruments for assessing the outcome variables were not specified.

Therefore, within two Special Interest Groups (SIGs) of the European Geriatric Medicine Society (EuGMS) the work towards developing a COS was initiated. As a first step in this process [12], we performed a scoping review to provide an overview of outcomes and their assessment methods used in nutritional intervention studies focused on the treatment of protein-energy malnutrition in older adults.

## Methods

This scoping review was conducted by interested members of the EuGMS SIG Nutrition, in close collaboration with the EuGMS SIG Systematic Reviews and Meta-analysis. All methods were prespecified. The protocol is available upon request.

### Search strategy

One author (GT) developed the search strategy which was reviewed and commented by members of the study group. The final search consisted of keywords and text words and database-specific syntax. References were retrieved from Medline (via Ovid), Embase (via Ovid), Cumulative Index to Nursing and Allied Health Literature (CINAHL via EbscoHost) and the Cochrane Central Register of Controlled Trials (CENTRAL via the Cochrane Library) from database inception through March 9, 2020. The full search strategies are shown in Supplementary Table S1. After removing duplicates, references of all retrieved items were uploaded in the systematic review web-application Covidence (Veritas Health Innovation, Melbourne, Australia; www.covidence.org).

### Screening

Screening was undertaken by all authors. Two authors independently screened each title and abstract for eligibility. When in doubt due to limited information, the reviewers were instructed to include the reference. In case of a discrepancy between two reviewers, the involved reviewers discussed their opinions and tried to reach agreement. If the conflict was still unsolved, one reviewer (MV) made the final decision. When for a single RCT a results paper was available, as well as a protocol paper or a trial registration, only the results paper was included.

Inclusion criteria:

- All languages
- Participants:
  o Age 65 years and above or, when the age range was not reported, a mean age of at least 70 years
  o With malnutrition (based on (i) a screening/assessment capturing multiple aspects of undernutrition, or (ii) BMI < 22 kg/m^2^, or (iii) involuntary weight loss (as defined by study authors)) *OR* at risk of malnutrition (based on a malnutrition screening tool)
  o All health conditions (e.g., an RCT conducted in hip fracture patients only was also included)
- All settings: community, hospital or long-term care/nursing home
- Interventions: nutritional intervention focused on increasing the intake of protein and/or energy (e.g., through dietetic counselling, provision of oral nutritional supplements (ONS) or protein supplements)
- Control condition: The contrast between the randomized groups is the increase in protein and/or energy (e.g., if the intervention contained ONS plus exercise and the control exercise only, the RCT was included)
- Study design: Randomized Controlled Trial (RCT), including quasi-randomized, cluster-randomized and randomized cross-over design
- Publication type: result paper, protocol paper, trial registration
- All outcomes

Exclusion criteria:

- Undernutrition defined as a micronutrient deficiency
- Nutritional interventions focused on adding micronutrients only
- Combined intervention, e.g., nutrition and exercise
- Conference abstracts, conference proceedings
- Not peer-reviewed publications (such as editorials)

Once all titles and abstracts were screened, full texts of the included references were uploaded in Covidence and screened for inclusion by two reviewers independently. When a reference was excluded, the reason for exclusion was indicated using the following fixed hierarchy to minimise potential conflicts: (1) full text not available, (2) wrong type of publication (e.g., conference abstract or conference proceeding), (3) wrong study type (e.g., not an intervention study or not randomized), (4) wrong population (e.g., not meeting age criterion or including well-nourished older adults), and (5) wrong contrast/comparator (e.g., intervention group included ONS plus exercise while control group received usual care). In case of discrepancies, the involved reviewers tried to reach agreement and if this was not feasible, a third reviewer (MV) made the final decision.

### Data extraction

For all included full texts, one author (MV) extracted the data using a standardised data extraction sheet. Two authors (NM and GT) each checked half of the extracted data. Disagreements were resolved through discussion between the two authors and consensus of the third author.

Extracted data included bibliographic information (first author, year of publication, country), setting (community, hospital, long-term care, mixed), sample description (general or specific patient group (e.g., hip fracture patients)), sample size of intervention group(s) and control group, age (mean age, age range or age inclusion criterion), method to assess (risk of) malnutrition, type of intervention and control condition, duration of the intervention and duration of the primary outcome follow-up, **primary and secondary outcome(s) and their assessment method**, level of potential conflict of interest (based on funding source(s), funding of used supplements, and potential authorship of funders), and type of paper (effect paper, protocol paper or trial registration).

During this phase, one RCT included from a trial registry was replaced by the results paper published after the search date [74].

### Descriptive synthesis

Characteristics of the studies and of the study sample, intervention type and control conditions, as well as funding information were categorized as indicated in Table 1.

**Table 1:** Main characteristics of the 60 included nutritional intervention RCTs to treat protein-energy malnutrition in older adults

Characteristic	Categories	Number of RCTs (%)
Publication year	≤ 2000	4 (7%)
	2001–2010	19 (32%)
	2011–2020	37 (61%)
Continent	Europe	40 (67%)
	Asia	9 (15%)
	North America	8 (13%)
	Australia	3 (5%)
Publication type	Results paper	50 (83%)
	Protocol paper	9 (15%)
	Trial registration	1 (2%)
Setting	Community^1^	26 (43%)
	Hospital	13 (22%)
	Long-term care	13 (22%)
	Mixed	7 (11%)
	NR	1 (2%)
Sample type	General	41 (68%)
	Specific patient group	18 (30%)
	NR	1 (2%)
Sample size (*n*)	≤ 50	14 (23%)
	51–100	20 (33%)
	101–200	21 (35%)
	200 +	4 (7%)
	NR	1 (2%)
Age (y)	65 + or > 65	37 (62%)
	70 + or > 70	9 (15%)
	Other	14 (23%)
Malnutrition status	At risk of malnutrition only	16 (27%)
	Malnourished only	4 (7%)
	Combination	40 (66%)
Intervention type	ONS	32 (54%)
	Dietary counselling	15 (25%)
	Dietary counselling + ONS	9 (15%)
	Protein supplement	2 (3%)
	Other	2 (3%)
Control	Usual care	37 (62%)
	Placebo ONS	5 (8%)
	Different type of ONS	5 (8%)
	Dietary counselling	4 (7%)
	Written information	4 (7%)
	Home visit(s)	5 (8%)
Intervention duration (w)	≤ 8	16 (27%)
	9–12	20 (33%)
	13–26	11 (18%)
	27–52	2 (3%)
	> 52	0 (0%)
	Other^2^	4 (7%)
	NR	7 (12%)
Follow-up duration (w)	≤ 8	9 (15%)
	9–12	25 (42%)
	13–26	14 (23%)
	27–52	8 (13%)
	> 52	2 (3%)
	Other	1 (2%)
	NR	1 (2%)
Study funding	Government and/or university	27 (45%)
	Government and/or university, with supplements provided by industry	3 (5%)
	(Co)funding by industry (and supplements provided by industry)	15 (25%)
	(Co)funding by industry and industry employee is (co)author	9 (15%)
	NR	6 (10%)

*NR* not reported. *ONS*  Oral Nutritional Supplement. *Y* years, w weeks

^1^Of which *n* = 11 just after hospital discharge, and *n* = 3 with home care

^2^Until hospital discharge or during chemotherapy treatment

For clarity reasons, several outcomes were categorized into an outcome domain: body circumference (including calf, thigh and mid-upper arm circumference), skinfold (including triceps, sub-scapula, supra-iliac and abdominal skinfold) and blood marker (including a wide variety of markers).

Outcomes were considered primary outcomes, when: (1) they were listed as primary outcomes in the article, or (2) they were not listed as primary outcomes but a power calculation for those outcomes was included. In case both criterion 1 and 2 could not be applied, all outcomes were considered as primary outcomes. The distinction between primary and secondary outcomes was made to highlight which outcomes were considered most critical by the investigators, as this information can be of potential help in the next steps of establishing a COS. In addition, by making this distinction we could also explore whether the heterogeneity in outcomes was potentially smaller for primary outcomes compared to secondary outcomes and whether the most frequently used outcomes across all RCTs were included as primary outcomes only.

The frequency of outcomes and outcome domains used in the included RCTs was determined, as well as the frequency of primary outcomes and outcome domains. In addition, the percentage of RCTs using a specific outcome (domain) was calculated. These analyses were repeated stratified by setting: community, hospital, long-term care, and other (i.e., RCTs with a mixed-setting or when no information about the setting was provided).

## Results

**Fig. 1:**
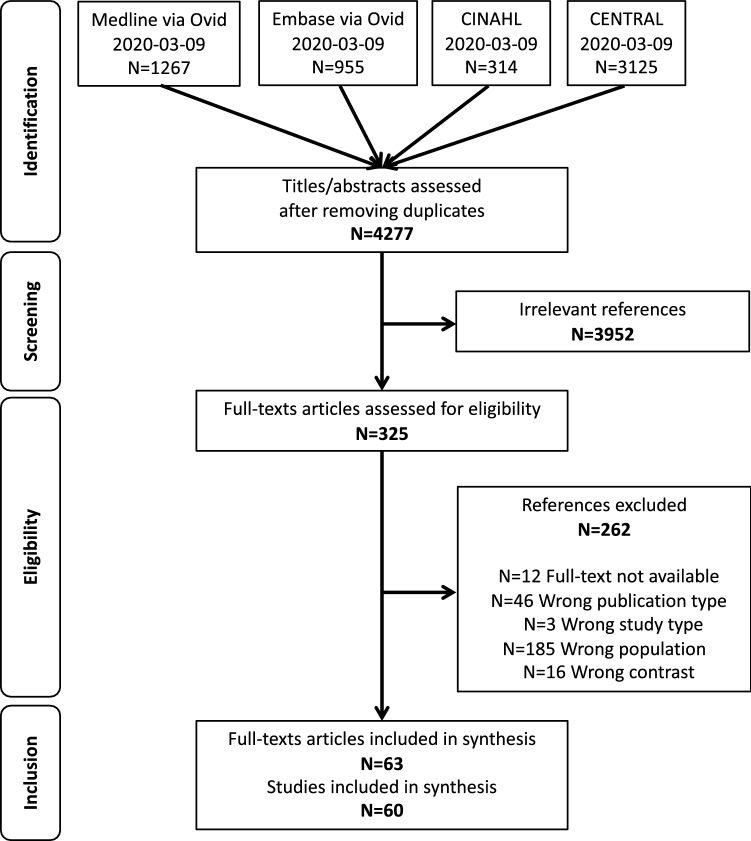
Flowchart

From the 4277 identified references, 325 full articles were screened, and 63 articles [13–75] describing 60 RCTs were included in our review (Fig. 1).

Table 1 describes the main characteristics of the included RCTs. The main characteristics of each individual included RCT are shown in supplementary Table S2.

The majority of the included RCTs was conducted in Europe (67%), published in the past 10 years (62%) and included community-dwelling older adults (43%). Most RCTs used age > 65 years as the inclusion criterion (62%) and recruited a combination of older adults with malnutrition and those at risk (67%). For the inclusion criteria, assessment of nutritional status was mostly based on the MNA, either assessed alone (n = 17; 28%) or in combination with some other measurements (n = 11; 18%) such as BMI, weight loss or albumin concentration. Low BMI was used as the sole criterion in 4 RCTs (7%), or in combination with other measurements in 14 RCTs (23%). Recent weight loss was mostly used in combination with other measurements (n = 19; 32%) and only rarely as single criterion (n = 1; 2%).

Sample size was mainly between 50 and 200 (68%) and the intervention lasted 12 weeks or less for most RCTs (60%). For seven RCTs the intervention duration was not reported (7%). Most RCTs provided either ONS or dietary counselling as the nutritional intervention (78%), and most included a control group receiving usual care (62%). For two RCTs (3%) providing dietary counselling only it was explicitly stated that this could include the prescription of ONS when deemed necessary by the health care professional involved. For nine RCTs (15%) dietary counselling was combined with daily ONS for all older adults throughout the whole intervention period (the dietary counselling + ONS category).

## Outcomes

**Fig. 2:**
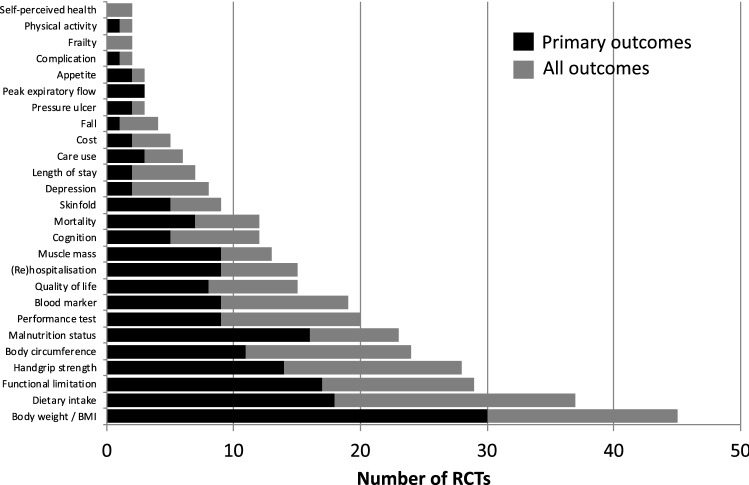
Frequency of the outcomes and outcome domains reported in at least two randomized controlled trials

The outcomes assessed in each of the 60 RCTs can be found in supplementary Table S3. Five RCTs use a single outcome only, while most RCTs had multiple outcomes. The frequency of the outcomes and outcome domains used in all included RCTs is shown in supplementary Table S4. Figure 2 shows the frequency of the outcomes and outcome domains reported in at least two RCTs. The frequency of these outcomes and outcome domains used as primary outcome is also indicated in Fig. 2. Across all RCTs, the top five of most frequently used outcomes or outcome domains included body weight/BMI, dietary intake, functional limitation, handgrip strength and body circumference. When only primary outcomes were considered, the top five was slightly different: body weight/BMI, dietary intake, functional limitation, malnutrition status and handgrip strength. The most frequently used outcomes or outcome domains were about equally included as a primary or secondary outcome, with the exception of body weight/BMI which was mostly included as a primary outcome (66.7%) in the included RCTs.

The top ten of most frequently used outcomes and outcome domains differed by setting (Table 2). Some outcomes were not, or almost never, included in a specific setting, such as cost in the community setting, blood marker and quality of life in the hospital setting, and functional performance, (re)hospitalization, muscle mass and mortality in the long-term care setting (Table S4).

**Table 2:** Top ten of most frequently used outcomes and outcome domains per setting

	Community (*n* = 26)	Hospital (*n* = 13)	Long-term care (*n* = 13)	Mixed or not reported (*n* = 8)
1	Body weight/BMI (*n* = 19; 73%)	Body weight/BMI (*n* = 9; 69%)	Body weight/BMI (*n* = 13; 100%)	Functional limitation (*n* = 6; 75%)
2	Dietary intake (*n* = 17; 65%)	Body circumference (*n* = 7; 54%)	Dietary intake (*n* = 12; 92%)	Body weight/BMI (*n* = 4; 50%)
3	Handgrip strength (*n* = 15; 58%)	Dietary intake (*n* = 5; 38%)	Malnutrition status (*n* = 8; 62%)	Malnutrition status (*n* = 4; 50%)
4	Functional limitation (*n* = 15; 58%)	Hangrip strength (*n* = 5; 38%)	Handgrip strength (*n* = 6; 46%)	Blood marker (*n* = 4; 50%)
5	Functional performance (*n* = 13; 50%)	Malnutrition status (*n* = 4; 31%)	Body circumference (*n* = 6; 46%)	Re (hospitalization) (*n* = 4; 50%)
6	Body circumference (*n* = 10; 38%)	Functional performance (*n* = 4; 31%)	Functional limitation (*n* = 5; 38%)	Dietary intake (*n* = 3; 38%)
7	(Re) hospitalization (*n* = 9; 35%)	Muscle mass (*n* = 4; 31%)	Blood marker (*n* = 5; 38%)	Mortality (*n* = 3; 38%)
8	Quality of life (*n* = 9; 35%)	Length of stay (*n* = 4; 31%)	Quality of life (*n* = 4; 31%)	Handgrip strength (*n* = 2; 25%)
9	Blood marker (*n* = 8; 31%)	Skinfolds (*n* = 3; 23%)	Appetite (*n* = 3; 31%)	Muscle mass (*n* = 2; 25%)
10	Malnutrition status (*n* = 7; 27%)	Functional limitation (*n* = 3; 23%)	Functional performance (*n* = 2; 15%)	Cognition (*n* = 2; 25%)

Fifty-five out of the 60 RCTs had more than one outcome and therefore percentages add to more than 100% by setting

*BMI* body mass index

### Specific outcome variables included in outcome domains

Table 3 shows the specific variables included within the three defined outcome domains and the frequency of these variables in the selected RCTs.

**Table 3:** Overview and frequency of specific variables included in the three outcome domains: body circumference, blood marker and skinfold

Domain	Methodology	Number of RCTs
Body circumference (*n* = 24)	Mid-upper arm (MUAC)	19
	Calf	11
	Thigh	1
Blood marker^1^ (*n* = 19)	Albumin	11
	C-reactive protein (CRP)	7
	Total cholesterol	7
	Pre-albumin	5
	Haemoglobin	5
	Vitamin D (25(OH)D)	5
	Transferrin	4
	Insulin-like growth factor-I (IGF-I)	4
	Lymphocyte count	4
	LDL cholesterol	4
	Total Blood cell count	3
	HDL cholesterol	3
	Haematocrit	3
	Triglycerides	3
	White blood cell count (WBC)	2
	Prognostic inflammatory nutritional index (PINI)	2
	Electrolytes	2
	Creatinine	2
	Calcium	2
	Fasting glucose	2
	Vitamin B12	2
	Folic acid	2
	Zinc	2
Skinfold (*n* = 9)	Triceps	9
	Sub-scapula	2
	Supra-iliac	1
	Abdominal	1

*LDL* low-density lipoprotein; *HDL* high-density lipoprotein

^1^Only blood markers that were assessed at least in two RCTs are included in the table. All included blood markers can be found in table S3

### Assessment methods of outcomes

Table 4 shows the methods used to assess outcomes. Outcomes used in a minimum of 10 RCTs are listed in Table 4, while the assessment method for all other outcomes used in the RCTs are shown in table S3. For some outcomes there seemed to be a clear preference for a certain assessment method (e.g., dietary records or 24-h dietary recalls to assess dietary intake, the Barthel index to assess functional limitations, and the MNA to assess malnutrition status), while for other outcomes there was a greater variation in assessment methods used (e.g., the instruments used for measuring handgrip strength or test used to assess functional performance). The variation in handgrip strength instruments results in variation in the measurement unit (kPA or kg) and variation in the used protocol to test handgrip strength, as some dynamometers can only be used with the arm hanging down instead of at a 90-degree angle. In some RCTs multiple assessment methods were used for a certain outcome.

**Table 4:** Overview and frequency of the methodology used to assess outcomes

Outcome	Methodology	Number of RCTs
Dietary intake (*n* = 37)	Dietary records	12
	24-h dietary recalls	11
	24-h dietary recalls or dietary records	2
	Food-frequency questionnaire	3
	Other (i.e., percentage of serving consumed, registration form, food intake protocol, 3-day count, and food chart)	5
	Not specified	4
Handgrip strength (*n* = 28)	JAMAR dynamometer	5
	Takei dynamometer	4
	Martin vigorimeter	3
	Smedley hand dynamometer	2
	Harpenden dynamometer	1
	Digimax dynamometer	1
	MSD dynamometer	1
	Tanita dynamometer	1
	Vital sign TM dynamometer	1
	SAEHAN dynamometer	1
	Not specified	7
Functional limitation (*n* = 29)	Barthel Activities of daily living (ADL) index	17
	Katz index of Independence in ADL	2
	Morton Mobility Index (DEMMI)	2
	Lawton Instrumental ADL (IADL) scale	2
	Avlund mobility-tiredness scale (Mob-T)	2
	Self-reported Disability Score	1
	Functional Independence Measure (FIM)	1
	Functional Assessment Screening Tool (FAST)	1
	Consumer Assessments Study Interview Battery (CAS)	1
	Not specified	3
Malnutrition status (*n* = 23)	Mini Nutritional Assessment (MNA)	13
	Mini Nutritional Assessment Short-Form (MNA-SF)	6
	Malnutrition Universal Screening Tool (MUST)	1
	Subjective Global Assessment (SGA)	1
	DETERMINE	1
	Not specified	2
Functional performance (*n* = 20)	Gait speed	7
	Timed Up and Go (TUG)	6
	Short Physical Performance Battery (SPPB)	5
	30 s chair stands	5
	1-leg stand	1
Quality of life (*n* = 15)	36-item Short Form Health Survey (SF-36)	7
	EuroQol (EQ-5D)	5
	QUALIDEM	1
	Dartmouth Primary Care Cooperative Information chart (COOP)	1
	Not specified	3
Muscle mass (*n* = 13)	Dual-energy X-ray absorptiometry (DXA)	8
	Bio-electrical impedance / bio-impedance spectroscopy	6
	Anthropometry (combination of mid-upper arm circumference and skinfold to derive muscle circumference)	3
	Deuterium oxide dilution	1
	Not specified	1

## Discussion

This scoping review was performed to provide a systematic overview of outcomes used in nutritional intervention studies focused on the treatment of protein-energy malnutrition in older adults. The review shows a large variation in used outcomes, not only across settings but also within a certain setting. Furthermore, a large variation in the methods used to assess these outcomes was observed for many outcomes. These results confirm the need for developing setting-specific COS for malnutrition intervention studies in older adults, to facilitate the future conduct of meta-analyses investigating the effectiveness of nutritional interventions as basis for evidence-based recommendations for clinical practice.

The selection of a study outcome is influenced by many factors, including relevance and responsiveness to treatment according to the researchers involved, local equipment (e.g., presence of a DXA scanner), local data access (e.g., access to standardized data from electronic patient files or mandatory assessments needed for reimbursement), time, costs and expertise available, or demands of the study funder. The most frequently used outcomes according to this review, should therefore not be viewed as the outcomes considered most relevant, most feasible or most responsive to treatment in the different settings. Further research is needed to determine which outcomes are considered most relevant and feasible for each specific setting.

The majority (92%) of the included RCTs had multiple outcomes, reflecting the breath of effects expected by a nutritional intervention. A direct effect of the intervention on dietary intake was evaluated in 62% of the RCTs, and its subsequent effect on body weight or malnutrition status was evaluated in 75% and 38% of the RCTs. A better nutritional status induced by the intervention may lead to many functional and clinical improvements, which might explain the wide variation in outcomes observed across RCTs. Thirty-three RCTs (55%) defined primary and secondary outcomes, while five RCTs (8%) defined a single outcome. These primary and single outcomes are likely to be considered most relevant by the researchers involved. However, the frequency pattern of the primary and single outcomes was fairly similar to the frequency pattern of all outcomes, suggesting that there were no specific outcomes more likely to be defined as primary outcome(s).

The overview of used outcomes obtained in this review will serve as an important basis for the next steps in developing a COS [12], including a web-based survey using a Delphi approach to rank the identified outcomes in the current study based on specific criteria. The survey will be distributed to researchers involved in malnutrition research in older adults as well as to health care professionals such as geriatricians and dieticians treating older adults with malnutrition, to avoid discipline-related biases [76]. A further step will include research among (malnourished) older adults in different settings to identify outcomes that are considered most relevant to them. In a final step, a setting-specific COS will be developed and published.

In performing the current scoping review, some additional observations were made that are of interest. First, the included RCTs show a large variation in defining malnutrition as the inclusion criterion for recruitment. As we applied specific inclusion criteria for the assessment of malnutrition for our review, the actual variation across RCTs is probably even larger. Recent efforts to reach global consensus on how malnutrition should be assessed and defined [77, 78] most likely will contribute to a greater overlap in these methods in the future, also supporting future meta-analyses. Second, in 18 RCTs (30%) no primary outcome was defined nor was a power calculation for a relevant outcome provided, increasing the risk for underpowered studies. This observation also highlights the need for future meta-analyses in this field. Third, a potential conflict of interest was identified in 27 included RCTs (45%) as nutritional products and/or funding was provided by industry, or employees of industry funders were included as authors. For six RCTs (10%) no information regarding funding was provided. These last two observations could indicate an increased risk of bias in several nutritional intervention studies.

A strength of our scoping review is the very strict methodology used in searching and reviewing the literature, extracting the data and reporting the results (Table S5) [79]. Furthermore, the complementary expertise of the authors has strengthened the review process. Another strength is that we included trial protocols in our review to ensure including outcomes of recently designed RCTs, as outcomes may vary over time for example due to recent scientific insights or the development of new assessment methods. However, a limitation of including trial protocols is that they often lack specific information on the assessment methods used to measure study outcomes. In several RCTs no distinction was made between primary and secondary outcomes, suggesting that all outcomes were deemed equally critical and relevant by the investigators. For these RCTs, in the absence of further information, we considered all outcomes as primary outcomes, which may not have been a correct interpretation.

In conclusion, this scoping review highlights the wide variety of outcomes used in nutritional intervention studies conducted in malnourished older adults and those at risk. Furthermore, it shows that the most frequently used outcomes differ by setting and that some outcomes are not used in specific settings. Finally, for most outcomes the methods used to assess the outcome were heterogeneous. The information obtained in this scoping review provides the necessary basis for the next steps in developing a COS for nutritional intervention studies focused on the treatment of protein-energy malnutrition in older adults.

## Supplementary Information

Below is the link to the electronic supplementary material.

Supplementary file1 (PDF 248 KB)

Supplementary file2 (PDF 188 KB)

Supplementary file3 (DOCX 26 KB)

Supplementary file4 (PDF 81 KB)

Supplementary file5 (DOCX 107 KB)
