# Supplementary material for: Towards developing a Core Outcome Set for malnutrition intervention studies in older adults: a scoping review to identify frequently used research outcomes
Source: Eur Geriatr Med. 2022 Mar 12;13(4):867–79. doi: 10.1007/s41999-022-00617-5 (PMC9378339; doi:10.1007/s41999-022-00617-5)
Supplement: Supplementary file 1 — Supplementary file1 (PDF 248 KB) [file 41999_2022_617_MOESM1_ESM.pdf]

TABLE S1. Search strategy in the four databases

Database: Ovid MEDLINE(R) ALL 1946 to March 06, 2020
Search date: 2020-03-09

#	Searches	Results
1	Protein-Energy Malnutrition/	7261
2	Malnutrition/	13201
3	malnutrition.kf,tw.	39374
4	malnourish*.kf,tw.	10448
5	undernutrition.kw,tw.	7205
6	undernourish*.kf,tw.	3523
7	nutrition* risk.kf,tw.	2539
8	((poor* or low*) adj1 (nutrition* status or nourish*)).kw,tw.	1705
9	((improve* or optim*) adj1 nutrition* status).kw,tw.	1083
10	exp Aged/	3061800
11	Independent Living/	5338
12	Nursing Homes/	34286
13	Homes For The Aged/	13679
14	Long-Term Care/	25609
15	Residential Facilities/	5379
16	Housing for the Elderly/	1603
17	Veterans/	16297
18	Hospitals, Veterans/	6637
19	((old* or senior) adj1 (adult* or person* or subject* or people or man or men or woman or women or patient* or inpatient* or outpatient* or resident* or citizen*)).kf,tw.	470233
20	elderl*.kf,tw.	243583
21	eldes*.kf,tw.	777
22	geriat*.kf,tw.	63749
23	veteran*.kf,tw.	35431
24	((commun* or indepen*) adj1 (dwell* or livi*)).kf,tw.	29728
25	community-dwell*.kf,tw.	23009
26	nursing home*.kf,tw.	30001
27	((nurs* or longterm* or long-term* or ((long or longe*) adj1 term) or day or respit*) adj1 (care or carin*)).kf,tw.	66752
28	((nurs* or care or carin* or assist* or healthcare* or health-care* or longterm* or long-term* or ((long or longe*) adj1 term) or shelter* or residen* or retir* or rest or resti* or respit*) adj1 (house* or housi* or home* or institut* or facilit* or unit* or center or centers* or centre* or accommod* or livi*)).kf,tw.	265202
29	((advanc* or high*) adj1 age*).kw,tw.	39748
30	(super adj1 age*).kf,tw.	151
31	superage*.kf,tw.	35

32	super-age*.kf,tw.	126
33	centen*.kf,tw.	6283
34	centa*.kf,tw.	1664
35	nonagen*.kf,tw.	1405
36	octagen*.kf,tw.	50
37	sept?agen*.kf,tw.	373
38	supercenten*.kf,tw.	102
39	senium*.kf,tw.	114
40	65 year*.kf,tw.	81184
41	randomized controlled trial.pt.	501219
42	controlled clinical trial.pt.	93562
43	randomized.ab.	471808
44	placebo.ab.	205632
45	clinical trials as topic.sh.	190299
46	randomly.ab.	328476
47	trial.ti.	214362
48	41 or 42 or 43 or 44 or 45 or 46 or 47	1273079
49	exp animals/ not humans.sh.	4675679
50	48 not 49	1171121
51	or/1-9	60096
52	or/10-40	3710190
53	51 and 52 and 50	1267

Database: Embase 1988 to 2020 Week 10
Search date: 2020-03-09

#	Searches	Results
1	protein calorie malnutrition/	4255
2	malnutrition/	52988
3	malnutrition.ti,ab,kw.	48657
4	malnourish*.ti,ab,kw.	12030
5	undernutrition.ti,ab,kw.	8104
6	undernourish*.ti,ab,kw.	3477
7	nutrition* risk.ti,ab,kw.	4269
8	((poor* or low*) adj1 (nutrition* status or nourish*)).ti,ab,kw.	2460
9	((improve* or optim*) adj1 nutrition* status).ti,ab,kw.	1738
10	exp aged/	2760788
11	independent living/	4145
12	nursing home/	42955
13	home for the aged/	7993
14	long term care/	121012
15	residential home/	5981
16	veteran/	24229
17	((old* or senior) adj1 (adult* or person* or subject* or people or man or men or woman or women or patient* or inpatient* or outpatient* or resident* or citizen*)).ti,ab,kw.	594488
18	elderl*.ti,ab,kw.	321281
19	eldes*.ti,ab,kw.	1055
20	geriat*.ti,ab,kw.	72305
21	veteran*.ti,ab,kw.	43322
22	((commun* or indepen*) adj1 (dwell* or livi*)).ti,ab,kw.	37920
23	community-dwell*.ti,ab,kw.	29402
24	nursing home*.ti,ab,kw.	34630
25	((nurs* or longterm* or long-term* or ((long or longe*) adj1 term) or day or respit*) adj1 (care or carin*)).ti,ab,kw.	69788
26	((nurs* or care or carin* or assist* or healthcare* or health-care* or longterm* or long-term* or ((long or longe*) adj1 term) or shelter* or residen* or retir* or rest or resti* or respit*) adj1 (house* or housi* or home* or institut* or facilit* or unit* or center or centers* or centre* or accommod* or livi*)).ti,ab,kw.	355537
27	((advanc* or high*) adj1 age*).ti,ab,kw.	53984
28	(super adj1 age*).ti,ab,kw.	210
29	superage*.ti,ab,kw.	48
30	super-age*.ti,ab,kw.	182
31	centen*.ti,ab,kw.	5027
32	centa*.ti,ab,kw.	2904
33	nonagen*.ti,ab,kw.	1954
34	octagen*.ti,ab,kw.	117

35	sept?agen*.ti,ab,kw.	506
36	supercenten*.ti,ab,kw.	119
37	senium*.ti,ab,kw.	89
38	65 year*.ti,ab,kw.	126331
39	(random$ or placebo$ or single blind$ or double blind$ or triple blind$).ti,ab.	1565463
40	retracted article/	9180
41	or/39-40	1574232
42	(animal$ not human$).sh,hw.	3453220
43	(book or conference paper or editorial or letter or review).pt. not exp randomized controlled trial/	4725541
44	(random sampl$ or random digit$ or random effect$ or random survey or random regression).ti,ab. not exp randomized controlled trial/	107788
45	41 not (42 or 43 or 44)	1197705
46	or/1-9	81407
47	or/10-38	3671011
48	46 and 47 and 45	1489
49	limit 48 to embase	955

RCT filter: https://bestpractice.bmj.com/info/toolkit/learn-ebm/study-design-search-filters/

Database: CINAHL via Ebscohost
Search date: 2020-03-09

EBSCOhost Monday, March 09, 2020 11:54:17 AM

#	Query	Limiters/Expanders	Last Run Via	Results
S1	MH Protein-Energy Malnutrition	Expanders - Apply equivalent subjects Search modes - Boolean/Phrase	Interface - EBSCOhost Research Databases Search Screen - Advanced Search Database - CINAHL	832
S2	MH Malnutrition	Expanders - Apply equivalent subjects Search modes - Boolean/Phrase	Interface - EBSCOhost Research Databases Search Screen - Advanced Search Database - CINAHL	7,760
S3	TI malnutrition OR AB malnutrition	Expanders - Apply equivalent subjects Search modes - Boolean/Phrase	Interface - EBSCOhost Research Databases Search Screen - Advanced Search Database - CINAHL	10,526
S4	TI malnourish* OR AB malnourish*	Expanders - Apply equivalent subjects Search modes - Boolean/Phrase	Interface - EBSCOhost Research Databases Search Screen - Advanced Search Database - CINAHL	2,693
S5	TI undernutrition OR AB undernutrition	Expanders - Apply equivalent subjects	Interface - EBSCOhost	1,918

		Search modes - Boolean/Phrase	Research Databases Search Screen - Advanced Search Database - CINAHL	
S6	TI undernourish* OR AB undernourish*	Expanders - Apply equivalent subjects Search modes - Boolean/Phrase	Interface - EBSCOhost Research Databases Search Screen - Advanced Search Database - CINAHL	762
S7	TI nutrition* risk OR AB nutrition* risk	Expanders - Apply equivalent subjects Search modes - Boolean/Phrase	Interface - EBSCOhost Research Databases Search Screen - Advanced Search Database - CINAHL	3,813
S8	TI ((poor* or low*) N1 (nutrition* status or nourish*)) OR AB ((poor* or low*) N1 (nutrition* status or nourish*))	Expanders - Apply equivalent subjects Search modes - Boolean/Phrase	Interface - EBSCOhost Research Databases Search Screen - Advanced Search Database - CINAHL	656
S9	TI ((improve* or optim*) N1 nutrition* status) OR AB ((improve* or optim*) N1 nutrition* status)	Expanders - Apply equivalent subjects Search modes - Boolean/Phrase	Interface - EBSCOhost Research Databases Search Screen - Advanced Search Database - CINAHL	840
S10	MH Aged+	Expanders - Apply equivalent subjects Search modes - Boolean/Phrase	Interface - EBSCOhost Research Databases Search Screen	761,776

			- Advanced Search Database - CINAHL	
S11	MH Independent Living	Expanders - Apply equivalent subjects Search modes - SmartText Searching	Interface - EBSCOhost Research Databases Search Screen - Advanced Search Database - CINAHL	131
S12	MH Nursing Home	Expanders - Apply equivalent subjects Search modes - SmartText Searching	Interface - EBSCOhost Research Databases Search Screen - Advanced Search Database - CINAHL	200
S13	MH Homes For The Aged	Expanders - Apply equivalent subjects Search modes - SmartText Searching	Interface - EBSCOhost Research Databases Search Screen - Advanced Search Database - CINAHL	357
S14	MH Long-Term Care	Expanders - Apply equivalent subjects Search modes - SmartText Searching	Interface - EBSCOhost Research Databases Search Screen - Advanced Search Database - CINAHL	905
S15	MH Residential Facilities	Expanders - Apply equivalent subjects Search modes - Boolean/Phrase	Interface - EBSCOhost Research Databases Search Screen - Advanced Search	4,201

			Database - CINAHL	
S16	MH Housing for the Elderly	Expanders - Apply equivalent subjects Search modes - Boolean/Phrase	Interface - EBSCOhost Research Databases Search Screen - Advanced Search Database - CINAHL	3,265
S17	MH Veterans	Expanders - Apply equivalent subjects Search modes - Boolean/Phrase	Interface - EBSCOhost Research Databases Search Screen - Advanced Search Database - CINAHL	14,948
S18	MH Hospitals, Veterans/	Expanders - Apply equivalent subjects Search modes - Boolean/Phrase	Interface - EBSCOhost Research Databases Search Screen - Advanced Search Database - CINAHL	4,780
S19	TI ((old* or senior) N1 (adult* or person* or subject* or people or man or men or woman or women or patient* or inpatient* or outpatient* or resident* or citizen*)) OR AB ((old* or senior) N1 (adult* or person* or subject* or people or man or men or woman or women or patient* or inpatient* or outpatient* or resident* or citizen*))	Expanders - Apply equivalent subjects Search modes - Boolean/Phrase	Interface - EBSCOhost Research Databases Search Screen - Advanced Search Database - CINAHL	118,283
S20	TI elderl* OR AB elderl*	Expanders - Apply equivalent subjects Search modes - Boolean/Phrase	Interface - EBSCOhost Research Databases Search Screen - Advanced Search	81,449

			Database - CINAHL	
S21	TI eldes* OR AB eldes*	Expanders - Apply equivalent subjects Search modes - Boolean/Phrase	Interface - EBSCOhost Research Databases Search Screen - Advanced Search Database - CINAHL	153
S22	TI geriat* OR AB geriat*	Expanders - Apply equivalent subjects Search modes - Boolean/Phrase	Interface - EBSCOhost Research Databases Search Screen - Advanced Search Database - CINAHL	24,014
S23	TI veteran* OR AB veteran*	Expanders - Apply equivalent subjects Search modes - Boolean/Phrase	Interface - EBSCOhost Research Databases Search Screen - Advanced Search Database - CINAHL	19,691
S24	TI (commun* or indepen*) N1 (dwell* or livi*) OR AB (commun* or indepen*) N1 (dwell* or livi*)	Expanders - Apply equivalent subjects Search modes - Boolean/Phrase	Interface - EBSCOhost Research Databases Search Screen - Advanced Search Database - CINAHL	19,583
S25	TI community-dwell* OR AB community-dwell*	Expanders - Apply equivalent subjects Search modes - Boolean/Phrase	Interface - EBSCOhost Research Databases Search Screen - Advanced Search Database - CINAHL	14,519

S26	TI nursing home* OR AB nursing home*	Expanders - Apply equivalent subjects Search modes - Boolean/Phrase	Interface - EBSCOhost Research Databases Search Screen - Advanced Search Database - CINAHL	23,967
S27	TI (nurs* or longterm* or long-term* or ((long or longe*) N1 term) or day or respit*) N1 (care or carin*) OR AB (nurs* or longterm* or long-term* or ((long or longe*) N1 term) or day or respit*) N1 (care or carin*)	Expanders - Apply equivalent subjects Search modes - Boolean/Phrase	Interface - EBSCOhost Research Databases Search Screen - Advanced Search Database - CINAHL	68,755
S28	TI (nurs* or care or carin* or assist* or healthcare* or health-care* or longterm* or long-term* or ((long or longe*) N1 term) or shelter* or residen* or retir* or rest or resti* or respit*) N1 (house* or housi* or home* or institut* or facilit* or unit* or center or centers* or centre* or accommod* or livi*) OR AB (nurs* or care or carin* or assist* or healthcare* or health-care* or longterm* or long-term* or ((long or longe*) N1 term) or shelter* or residen* or retir* or rest or resti* or respit*) N1 (house* or housi* or home* or institut* or facilit* or unit* or center or centers* or centre* or accommod* or livi*)	Expanders - Apply equivalent subjects Search modes - Boolean/Phrase	Interface - EBSCOhost Research Databases Search Screen - Advanced Search Database - CINAHL	157,835
S29	TI (advanc* or high*) N1 age* OR AB (advanc* or high*) N1 age*	Expanders - Apply equivalent subjects Search modes - Boolean/Phrase	Interface - EBSCOhost Research Databases Search Screen - Advanced Search Database - CINAHL	13,651
S30	TI super N1 age* OR AB super N1 age*	Expanders - Apply equivalent subjects	Interface - EBSCOhost	62

		Search modes - Boolean/Phrase	Research Databases Search Screen - Advanced Search Database - CINAHL	
S31	TI superage* OR AB superage*	Expanders - Apply equivalent subjects Search modes - Boolean/Phrase	Interface - EBSCOhost Research Databases Search Screen - Advanced Search Database - CINAHL	9
S32	TI super-age* OR AB super-age*	Expanders - Apply equivalent subjects Search modes - Boolean/Phrase	Interface - EBSCOhost Research Databases Search Screen - Advanced Search Database - CINAHL	52
S33	TI centen* OR AB centen*	Expanders - Apply equivalent subjects Search modes - Boolean/Phrase	Interface - EBSCOhost Research Databases Search Screen - Advanced Search Database - CINAHL	1,526
S34	TI centa* OR AB centa*	Expanders - Apply equivalent subjects Search modes - Boolean/Phrase	Interface - EBSCOhost Research Databases Search Screen - Advanced Search Database - CINAHL	248
S35	TI nonagen* OR AB nonagen*	Expanders - Apply equivalent subjects Search modes - Boolean/Phrase	Interface - EBSCOhost Research Databases Search Screen	514

			- Advanced Search Database - CINAHL	
S36	TI octagen* OR AB octagen*	Expanders - Apply equivalent subjects Search modes - Boolean/Phrase	Interface - EBSCOhost Research Databases Search Screen - Advanced Search Database - CINAHL	11
S37	TI septagen* OR AB septagen*	Expanders - Apply equivalent subjects Search modes - Boolean/Phrase	Interface - EBSCOhost Research Databases Search Screen - Advanced Search Database - CINAHL	1
S38	TI septuagen* OR AB septuagen*	Expanders - Apply equivalent subjects Search modes - Boolean/Phrase	Interface - EBSCOhost Research Databases Search Screen - Advanced Search Database - CINAHL	100
S39	TI supercenten* OR AB supercenten*	Expanders - Apply equivalent subjects Search modes - Boolean/Phrase	Interface - EBSCOhost Research Databases Search Screen - Advanced Search Database - CINAHL	59
S40	TI senium* OR AB senium*	Expanders - Apply equivalent subjects Search modes - Boolean/Phrase	Interface - EBSCOhost Research Databases Search Screen - Advanced Search	4

			Database - CINAHL	
S41	TI 65 year* OR AB 65 year*	Expanders - Apply equivalent subjects Search modes - Boolean/Phrase	Interface - EBSCOhost Research Databases Search Screen - Advanced Search Database - CINAHL	24,015
S42	MH randomized controlled trials	Expanders - Apply equivalent subjects Search modes - Boolean/Phrase	Interface - EBSCOhost Research Databases Search Screen - Advanced Search Database - CINAHL	91,071
S43	MH double-blind studies	Expanders - Apply equivalent subjects Search modes - Boolean/Phrase	Interface - EBSCOhost Research Databases Search Screen - Advanced Search Database - CINAHL	43,581
S44	MH single-blind studies	Expanders - Apply equivalent subjects Search modes - Boolean/Phrase	Interface - EBSCOhost Research Databases Search Screen - Advanced Search Database - CINAHL	13,167
S45	MH random assignment	Expanders - Apply equivalent subjects Search modes - Boolean/Phrase	Interface - EBSCOhost Research Databases Search Screen - Advanced Search Database - CINAHL	57,734

S46	MH pretest-posttest design	Expanders - Apply equivalent subjects Search modes - Boolean/Phrase	Interface - EBSCOhost Research Databases Search Screen - Advanced Search Database - CINAHL	39,791
S47	MH cluster sample	Expanders - Apply equivalent subjects Search modes - Boolean/Phrase	Interface - EBSCOhost Research Databases Search Screen - Advanced Search Database - CINAHL	4,068
S48	TI (randomised OR randomized)	Expanders - Apply equivalent subjects Search modes - Boolean/Phrase	Interface - EBSCOhost Research Databases Search Screen - Advanced Search Database - CINAHL	98,061
S49	AB (random*)	Expanders - Apply equivalent subjects Search modes - Boolean/Phrase	Interface - EBSCOhost Research Databases Search Screen - Advanced Search Database - CINAHL	283,631
S50	TI (trial)	Expanders - Apply equivalent subjects Search modes - Boolean/Phrase	Interface - EBSCOhost Research Databases Search Screen - Advanced Search Database - CINAHL	99,899
S51	MH (sample size) AND AB (assigned OR allocated OR control)	Expanders - Apply equivalent subjects	Interface - EBSCOhost Research	3,783

		Search modes - Boolean/Phrase	Databases Search Screen - Advanced Search Database - CINAHL	
S52	MH (placebos)	Expanders - Apply equivalent subjects Search modes - Boolean/Phrase	Interface - EBSCOhost Research Databases Search Screen - Advanced Search Database - CINAHL	11,547
S53	PT (randomized controlled trial)	Expanders - Apply equivalent subjects Search modes - Boolean/Phrase	Interface - EBSCOhost Research Databases Search Screen - Advanced Search Database - CINAHL	86,205
S54	AB (control W5 group)	Expanders - Apply equivalent subjects Search modes - Boolean/Phrase	Interface - EBSCOhost Research Databases Search Screen - Advanced Search Database - CINAHL	100,002
S55	MH (crossover design) OR MH (comparative studies)	Expanders - Apply equivalent subjects Search modes - Boolean/Phrase	Interface - EBSCOhost Research Databases Search Screen - Advanced Search Database - CINAHL	258,906
S56	AB (cluster W3 RCT)	Expanders - Apply equivalent subjects Search modes - Boolean/Phrase	Interface - EBSCOhost Research Databases Search Screen - Advanced	327

			Search Database - CINAHL	
S57	MH animals+	Expanders - Apply equivalent subjects Search modes - Boolean/Phrase	Interface - EBSCOhost Research Databases Search Screen - Advanced Search Database - CINAHL	86,382
S58	MH (animal studies)	Expanders - Apply equivalent subjects Search modes - Boolean/Phrase	Interface - EBSCOhost Research Databases Search Screen - Advanced Search Database - CINAHL	111,267
S59	TI (animal model*)	Expanders - Apply equivalent subjects Search modes - Boolean/Phrase	Interface - EBSCOhost Research Databases Search Screen - Advanced Search Database - CINAHL	2,857
S60	S57 OR S58 OR S59	Expanders - Apply equivalent subjects Search modes - Boolean/Phrase	Interface - EBSCOhost Research Databases Search Screen - Advanced Search Database - CINAHL	189,864
S61	MH (human)	Expanders - Apply equivalent subjects Search modes - Boolean/Phrase	Interface - EBSCOhost Research Databases Search Screen - Advanced Search Database - CINAHL	2,036,634

S62	S60 NOT S61	Expanders - Apply equivalent subjects Search modes - Boolean/Phrase	Interface - EBSCOhost Research Databases Search Screen - Advanced Search Database - CINAHL	166,851
S63	S42 OR S43 OR S44 OR S45 OR S46 OR S47 OR S48 OR S49 OR S50 OR S51 OR S52 OR S53 OR S54 OR S55 OR S56	Expanders - Apply equivalent subjects Search modes - Boolean/Phrase	Interface - EBSCOhost Research Databases Search Screen - Advanced Search Database - CINAHL	661,667
S64	S63 NOT S62	Expanders - Apply equivalent subjects Search modes - Boolean/Phrase	Interface - EBSCOhost Research Databases Search Screen - Advanced Search Database - CINAHL	632,904
S65	S1 OR S2 OR S3 OR S4 OR S5 OR S6 OR S7 OR S8 OR S9	Expanders - Apply equivalent subjects Search modes - Boolean/Phrase	Interface - EBSCOhost Research Databases Search Screen - Advanced Search Database - CINAHL	20,131
S66	S10 OR S11 OR S12 OR S13 OR S14 OR S15 OR S16 OR S17 OR S18 OR S19 OR S20 OR S21 OR S22 OR S23 OR S24 OR S25 OR S26 OR S27 OR S28 OR S29 OR S30 OR S31 OR S32 OR S33 OR S34 OR S35 OR S36 OR S37 OR S38 OR S39 OR S40 OR S41	Expanders - Apply equivalent subjects Search modes - Boolean/Phrase	Interface - EBSCOhost Research Databases Search Screen - Advanced Search Database - CINAHL	1,006,499
S67	S65 AND S66 AND S64	Expanders - Apply equivalent subjects	Interface - EBSCOhost Research	1,131

		Search modes - Boolean/Phrase	Databases Search Screen - Advanced Search Database - CINAHL	
S68	S65 AND S66 AND S64	Limiters - Exclude MEDLINE records Expanders - Apply equivalent subjects Search modes - Boolean/Phrase	Interface - EBSCOhost Research Databases Search Screen - Advanced Search Database - CINAHL	332
S69	S65 AND S66 AND S64	Academic Journals	Interface - EBSCOhost Research Databases Search Screen - Advanced Search Database - CINAHL	314

RCT Filter Glanville et al
https://onlinelibrary.wiley.com/doi/full/10.1111/hir.12251

Database: CENTRAL
Search date: 2020-03-09

ID Search Hits
#1 MeSH descriptor: [Protein-Energy Malnutrition] explode all trees 247
#2 MeSH descriptor: [Malnutrition] explode all trees 4058
#3 (malnutrition):ti,ab,kw 4290
#4 (malnourish*):ti,ab,kw 1312
#5 (undernutrition):ti,ab,kw 496
#6 (undernourish*):ti,ab,kw 346
#7 (nutrition* risk):ti,ab,kw 8492
#8 ((poor* or low*) NEAR/1 (nutrition* status or nourish*)):ti,ab,kw763
#9 ((improve* or optim*) NEAR/1 nutrition* status):ti,ab,kw 551
#10 MeSH descriptor: [Aged] explode all trees 1312
#11 MeSH descriptor: [Independent Living] explode all trees 379
#12 MeSH descriptor: [Nursing Homes] explode all trees 1293
#13 MeSH descriptor: [Homes for the Aged] explode all trees 604
#14 MeSH descriptor: [Long-Term Care] explode all trees 1107
#15 MeSH descriptor: [Residential Facilities] explode all trees 1684
#16 MeSH descriptor: [Housing for the Elderly] explode all trees 38
#17 MeSH descriptor: [Veterans] explode all trees 897
#18 MeSH descriptor: [Hospitals, Veterans] explode all trees 285
#19 ((old* or senior) NEAR/1 (adult* or person* or subject* or people or man or men or woman or women or patient* or inpatient* or outpatient* or resident* or citizen*)):ti,ab,kw 27834
#20 (elderl*):ti,ab,kw 46929
#21 (eldes*):ti,ab,kw 28
#22 (geriat*):ti,ab,kw 8116
#23 (veteran*):ti,ab,kw 5507
#24 ((commun* or indepen*) NEAR/1 (dwell* or livi*)):ti,ab,kw 5020
#25 (community-dwell*):ti,ab,kw3898
#26 (nursing home*):ti,ab,kw 6085
#27 ((nurs* or longterm* or long-term* or ((long or longe*) NEAR/1 term) or day or respit*) NEAR/1 (care or carin*)):ti,ab,kw 11584
#28 (((nurs* or care or carin* or assist* or healthcare* or health-care* or longterm* or long-term* or ((long or longe*) NEAR/1 term) or shelter* or residen* or retir* or rest or resti* or respit*) NEAR/1 (house* or housi* or home* or institut* or facilit* or unit* or center or centers* or centre* or accommod* or livi*))):ti,ab,kw 41084
#29 ((advanc* or high*) NEAR/1 age*):ti,ab,kw2342
#30 (super NEAR/1 age*):ti,ab,kw 7
#31 (superage*):ti,ab,kw 6
#32 (super-age*):ti,ab,kw 6
#33 (centen*):ti,ab,kw 40
#34 (centa*):ti,ab,kw 118
#35 (nonagen*):ti,ab,kw 32
#36 (octagen*):ti,ab,kw 0
#37 (septagen*):ti,ab,kw 0
#38 (septuagen*):ti,ab,kw 16
#39 (supercenten*):ti,ab,kw 0
#40 (senium*):ti,ab,kw 18
#41 (65 year*):ti,ab,kw 58429

#42 #1 OR #2 OR #3 OR #4 OR #5 OR #6 OR #7 OR #8 OR #9 15906
#43 #10 OR #11 OR #12 OR #13 OR #14 OR #15 OR #16 OR #17 OR #18 OR #19 OR #20 OR #21 OR #22 OR #23 OR #24 OR #25 OR #26 OR #27 OR #28 OR #29 OR #30 OR #31 OR #32 OR #33 OR #34 OR #35 OR #36 OR #37 OR #38 OR #39 OR #40 OR #41 160516
#44 #42 AND #43 3206
3125 Trials
