# Supplementary material for: Towards developing a Core Outcome Set for malnutrition intervention studies in older adults: a scoping review to identify frequently used research outcomes
Source: Eur Geriatr Med. 2022 Mar 12;13(4):867–79. doi: 10.1007/s41999-022-00617-5 (PMC9378339; doi:10.1007/s41999-022-00617-5)
Supplement: Supplementary file 2 — Supplementary file2 (PDF 188 KB) [file 41999_2022_617_MOESM2_ESM.pdf]

Table S2. Characteristics of the 60 included randomized controlled trials

Ref	Country	Setting	Sample	Age (y)	Malnutrition inclusion criteria	Sample size (I = intervention, C = control group)	Nutritional intervention	Control group	Duration intervention	Duration follow-up of primary outcome(s)	Funding source
32	USA	nursing home	Residents of nursing home and long-term care facility, mostly bedridden	Mean I=72, C=72	Malnourished (serum albumin<35 g/L or body weight >10% below the midpoint of the weight range recommended by an age-specific body weight table) or at risk (recent involuntary weight loss (>=2 kg in 2 w) or referral for inadequate dietary intake)	I=15, C=13	complete diet containing 24 en% protein (1060 kcal and 61 g protein per L) as tube feeding or meal replacement	complete diet containing 14 en% protein (1000 kcal and 37 g protein per L) as tube feeding or meal replacement	8 w	8 w	(Co)funding by industry and industry employee is (co)author
14	UK	hospital	Residents of care for the elderly wards with dementia at psychiatric teaching hospital	Mean I=69 men and 80 women, mean C=68 men and 79 women	BMI 15.1-19.9 kg/m^2	I=23, C=23	ONS	placebo ONS	12 w	12 w	(Co)funding by industry (and supplements provided by industry)
15	Germany	hospital and after discharge	Patients admitted to acute care ward of geriatric centre	75+	Malnourished (by clinical judgement of examining physician (reduced subcutaneous fatty tissue, prominent rib and shoulder bones observed, slack and flabby skinfolds at the backside and abdomen, BMI was considered when available))	I=35, C=37	ONS during hospitalization and after discharge	none	6 m after discharge	at discharge and 6 m after discharge	(Co)funding by industry (and supplements provided by industry)
16	France	nursing home	Residents of privately-run nursing homes	65+	At risk (MNA 17-23.5)	I=13, C=22	ONS	none	60 d	60 d	(Co)funding by industry and industry employee is (co)author
17	France	hospital	Hospitalized patients	69-90	Malnourished or at risk (weight loss >5% and/or albumin<38g/l)	I=17, C=6	ONS	none	10 d	10 d	(Co)funding by industry (and supplements

											provided by industry)
18	Denmark	nursing home	Nursing home residents	65+	Malnourished and at risk (MNA 17-23.5 and BMI<24 (group B and C), MNA<17 (group A)	I=8, C=8	homemade ONS	none	2 m	2 m	Government and/or university
19	Canada	community	Adults receiving long-term home-care services from local community service centres	>65	Malnourished and at risk ((a) weight loss >5% in past m, >7.5% in past 3 m, or >10% in the past 6 m, AND, BMI<27, or b) BMI<24)	I=41, C=42	ONS, home visit every 2 w to give nutritional counselling and encouragement to take supplements	no ONS but monthly home visits with small gift	16 w	16 w	(Co)funding by industry (and supplements provided by industry)
20	Belgium	hospital	Patients hospitalized in geriatric ward, admitted for acute conditions	75+	At risk (MNA-SF when <11, full form was done: MNA 17-23.5 included)	I=39, C=41	ONS	none	60 d	60 d	NR
21	UK	community	Community-dwelling women recruited through local GPs patient database, with osteoporosis at femoral neck and/or total hip	>70	At risk (BMI<=21)	I=36, C=35	some calcium and D, dietary advice and ONS	none	6 m	12 m	(Co)funding by industry (and supplements provided by industry)
22	UK	community (post discharge)	Patients to be discharged from hospital	65+	At risk (either: BMI<20, or BMI>=20 but <25 with documented evidence of weight loss of >=10% of body weight in the 6 m prior to the study period or >=5% in the 3 m prior to the study period)	I=51, C=49	ONS and dietetic counselling	none	8w	12 w and 24 w	(Co)funding by industry and industry employee is (co)author
23	France	geriatric wards and day centres	Patients with Alzheimer's' disease	65+	Malnourished or at risk (MNA<=23.5)	I=46, C=45	ONS	none	3 m	3 m and 6 m	(Co)funding by industry and industry employee is (co)author
24	UK	community/ hospital after discharge	Patients admitted with acute illness to general medical wards or	75+	Malnourished or at risk (BMI<=24 and TSF or MUAC below 10th percentile and/or weight loss >=5% during hospital stay)	I=66, C=70	ONS	none	8 w	12 w	Government and/or university, with supplements

			medicine for the elderly assessment wards and for whom discharge back to community was considered								provided by industry
25	Spain	nursing home	Patients with diagnosed Alzheimer's' disease DSM-IV criteria living in geriatric institution	>65	At risk (need a semi-solid or liquid diet and weight loss >5% in previous year)	I=24, C=29	Whole-formula diet based on lyophilized foods and snacks, nutritional advice	nutritional advice	3 m	3 m	(Co)funding by industry (and supplements provided by industry)
26	Norway	hospital + community	Medical inpatients/outpatients and district nurse patients able to walk	65+	At risk (BMI<20)	Enrolment n=23	Dietary advice and ONS	Dietary advice and recommendation of four meals per d	12 w	12 w	Government and/or university
27	Israel	hospital and after discharge	Patients admitted to internal medicine department at hospital	65+	At risk ('Among undernourished patients': no information regarding malnutrition status at in- and exclusion criteria)	Targeted n=180	I1: multi-disciplinary dietary intervention in hospital, I2: intensive intervention in community	none	NR	1 year following hospitalization	NR
28	Sweden	hospital and after discharge	Patients admitted to two wards at department of geriatric medicine	Mean I=85, C=85	At risk (MNA-SF<=10)	I=51, C=57	2 individualized counselling sessions by a dietitian + 3 phone contacts + diet advice + ONS + daily multivitamin supplement	Brief written dietary advice	4 m	4 m	(Co)funding by industry (and supplements provided by industry)
29	USA	hospital	Patients admitted to stroke services	Mean I=73, C=75	At risk (weight loss >=2.5% within 2 w after stroke onset)	I=58, C=58	ONS (240 kcal, 11 g protein)	ONS (127 kcal, 5 g protein)	Throughout the inpatient stay	at discharge	Government and/or university
30	Germany	nursing home	Frail nursing home residents	60-103	Malnourished and at risk (MNA<=23.5)	I=22, C=30	standard diet with protein- and energy enriched soups	standard diet	12 w	12 w	(Co)funding by industry (and supplements

							and sauces and 2 snacks				provided by industry)
31	Australia	community	Adults living independently in community	Mean men 77, women 77	At risk (MNA<24 and a BMI<22 or a self-reported weight loss of >=7.5% in the 3 m before enrolment)	I1=13, I2=11, C1=13, C2=12	I1: standard care and placebo testosterone and ONS. I2: standard care and testosterone and ONS	C1: standard care and placebo testosterone C2: standard care and testosterone	1 year	1 year	(Co)funding by industry (and supplements provided by industry)
32	Scotland, UK	community (hospital discharge)	Adults just discharged from the hospital	70+	Malnourished and at risk (BMI<24 and mid-arm muscle below 10th percentile or weight loss of 5% or more during hospital stay)	I=126, C=127	ONS (600 kcal/d)	ONS (200 kcal/d)	16 w	8 w and 16 w	Government and/or university, with supplements provided by industry
33	France	hospital	Patients with catabolic stress	mean I=82, C=79	Malnourished and at risk (MNA<24 and prognostic inflammatory nutritional index>=10)	I=13, C=13	ONS	none	2 w	2 w	(Co)funding by industry (and supplements provided by industry)
34, 35	Norway	hospital	Stroke patients from medical acute care ward hospital	>65	Malnourished and at risk (MUST, adapted with BMI cut-off <=20. Nutritional risk was either BMI<=20, or intentional weight loss of >=5% the previous 3-6 m, or poor nutritional intake for at least 5 d, or the risk of inadequate nutritional intake for the next 5 d)	I=58, C=66	Energy and protein enriched meals, or ONS, or enteral tube feeding according to needs calculated. Discharged with nutritional advice given by dietitian.	none	while in hospital	3 m	Government and/or university
36	USA	community	Homebound adults with either an acute illness or chronic condition	65+	Malnourished or at risk (not consuming enough calories to maintain body weight or weight loss of >5% over past 6 m)	Target n=104	Nutrition assessment and an in-home intervention with follow-up calls from a Registered Dietitian	nutrition assessment	NR	2 and 8 m	Government and/or university

							addresses risk for becoming under-nourished at multiple levels				
37	Australia	hospital	Female patients admitted to general hospital after a fracture	>70	Malnourished or at risk (moderate: MUAC<10th percentile for age and gender, or if pre-surgery serum albumin <=35 g/L; severe: both conditions were met)	I=23, C=21	ONS (475 kcal, 21g protein) in hospital and at home	A high protein diet (with high protein milk) in hospital (194 kcal, 11g protein)	40 d	40 d and 4 m	Government and/or university
38	Israel	during and after hospital	Patients admitted to internal medicine departments	65+	Malnourished and at risk (MNA-SF<10 or those who lost >10% of weight in the previous 6 m)	I1=78, I2=73, C=108	I1: Dietitian visit at hospital plus 3 home visits; I2: Dietitian visit at hospital. ONS as recommended by dietitian	Regular dietetic services	6 m	3 and 6 m	Government and/or university
39	South Korea	community	Frail older adults of low socioeconomic status	65+	Malnourished and at risk (MNA<24)	Enrolled n=87 enrolled	ONS	no ONS, monthly home visits	12 w	12 w	Government and/or university
40	Spain	community - home care	Patients of home care program carried out in primary care centres	>=65	At risk (MNA 17 to 23.5)	I=97, C=97	1 h education session for caregivers + monthly home visits up to 6 m + home visit at 12 m to provide individualized dietary advice	no nutritional intervention (one regular visit at baseline, 6 m and 12 m)	6 m	6 and 12 m	Government and/or university
41	Australia	hospital	Patients admitted to acute, geriatric medicine ward of hospital	mean I=83, C=83	Malnourished or at risk (MNA)	I=59, C=60	referral to clinical dietitian for nutritional intervention following a tailored malnutrition care plan	Usual nutrition care (dietitian not informed about screening result)	During hospital stay	6 m	(Co)funding by industry (and supplements provided by industry)
42	Denmark	community - post hospital	Discharged patients	65+	Malnourished or at risk (nutritional risk according to following criteria in the level 1 screen NRS-2002: BMI<20.5	I=73, C=79	12 w individualized nutritional counselling +	none	12 w	12 w	Government and/or university

					and/or weight loss in last 3 m and/or seriously ill)		GP follow-up visits in 3 home visits				
43	UK	residential care	Residents from residential care homes for older people ran by a charitable organization	70-105	Malnourished (BMI<18.5)	I=22, C=19	usual meals enriched with standard quantities of energy dense foods (50g double cream and 8g butter) plus a 250 ml milk drink in evening	usual meals	12 w	12 w	(Co)funding by industry and industry employee is (co)author
44	South Korea	community	Adults recruited from National Home Healthcare Services database, all whose families are below 120% of the national absolute poverty line, who could not walk 3m within 5 sec	65+	Malnourished or at risk (MNA<24)	I=43, C=44	ONS + home visit every 2 w by dietitian	no treatment or counselling, dietitian visit every month and small present	12 w	12 w	Government and/or university
45	USA	community - home care	Adults living at home and homebound who were receiving Medicare home health services	65+	Malnourished or at risk (consuming insufficient calories (caloric intake of 5% or more below the estimated energy requirement) and/or had a history of weight loss >=2.5% over 6 m)	I=18, C=16	Dietitian's home visit to improve caloric intake using self-management goal setting, and support calls dietitian at 1 w, 2 w and 4 w following the intervention	none	4 w	60 d	Government and/or university
46, 47	Netherlands	community	Adults recruited in primary care locations	65+	Malnourished or at risk (malnourished: MUAC<25 cm and /or self-report of >=4 kg weight loss in past 6 m; at risk:	I=72, C=74	dietetic treatment throughout the study	usual care + standard brochure about healthy eating habits	NR	3 m and 6 m	Government and/or university

					undernourished according to SNAQ65+)						
48	Germany	nursing home	Nursing home residents	Mean I=87, C=86	Malnourished or at risk (MNA<24, BMI<=22, low food intake according to nurses' perception, or weight loss of 5% or more in past 3 m or 10% or more in past 6 m)	I=45, C=42	ONS + encouragement by care staff	usual care (including ONS when prescribed by GP or provided by family members)	12 w	12 w	(Co)funding by industry and industry employee is (co)author
49	France	Community	Patients with lymphoma or carcinoma with indication for chemotherapy and Karnofsky index > 50%	70+	At risk (MNA 17-23.5)	I=169, C=167	Dietary advice complemented with ONS	none	6 times for 3 to 6 m according to chemotherapy duration	1 year	(Co)funding by industry (and supplements provided by industry)
50	Denmark	community - post-hospital	Discharged patients from geriatric medicine ward or orthopaedic surgery	70+	Malnourished and at risk (level 2 screening NRS-2002) and received ONS)	I=34, C=37	3 home visits by dietitian	none	8 w	12 w after discharge, (re-)hospitalizations after 30 d, 12 w and 6 m, mortality 12 w and 6 m.	Government and/or university
51	Germany	nursing home	Residents from catholic welfare organization operating nursing homes	65+	Malnourished and at risk (MNA<24, BMI<=22, low recent food intake or weight loss >=5% or 10% in the last 3 or 6 m, respectively)	I=45, C=42	ONS	Usual care, which could include ONS when prescribed by physician or provided by family members	12 w	12 w	(Co)funding by industry and industry employee is (co)author
52, 53	France	nursing home	Nursing home residents	>70	At risk (current prescription of home-made sweets enriched with milk proteins (and with weight loss in 4 w period before sweets) and/or liquid or creamy ONS, but who were diagnosed as malnourished)	I=88, C=87	8 cookies daily	none	6 w	6 w	Government and/or university
54	UK	care home	Care home residents	>65	Malnourished or at risk (without a dietetic-led plan, MUST score of 1 and higher, able to eat and drink)	I1=32, I2=29, C=32	I1: food-based intervention (care staff advice to increase	none	6 m	6 m	Government and/or university, with supplements

							intake); I2: ONS intervention				provided by industry
55	Sweden	residential care	Care home residents	65+	Malnourished and at risk (excluded are well-nourished according to MNA-SF 12-14 points and BMI>=28)	Cross-over: n=19 versus n=20	ONS	none	6 w	6 w	NR
56	USA	hospital and post-discharge	Patients with a primary diagnosis of CHF, AMI, PNA or COPD	65+	Malnourished and at risk (SGA class B or class C)	I=328, C=324	ONS (350 kcal, 20 g protein)	ONS (48 kcal, 0 g protein)	90 d after discharge	30 d, 60 d and 90 d	(Co)funding by industry and industry employee is (co)author
57	Netherlands	NR	NR	65+	Malnourished or at risk	Enrolled n=82	ONS (new product)	ONS (usual product)	12 w	12 w	Government and/or university
58	Denmark	community after discharge	Adults living independently and alone after hospitalisation	75+	Malnourished and at risk (MNA<24)	I1=73, I2=68, C=67	nutritional counselling after discharge by clinical dietitian, either in-person in home (I1) or by phone (I2)	none	4 w	8 w	NR
59	France	nursing home	Nursing home residents	70+	Malnourished and at risk (MNA <=23.5 or plasma pre-albumin <=0.2 g/L)	I1=35, I2=27, C=25	I1: enriched brioche; I2: ONS	usual breakfast	12 w	3 m	Government and/or university
60	USA	community - hospital discharge	Patients recruited at bedside at hospital acute care for Elders when discharged to a private residence or a facility where the patient was responsible for preparing own meals	65+	Malnourished and at risk (MNA<24)	I=12, C=12	book on smart food choices plus 3 meals per d for 10 d delivered at home	book on smart food choices	10 d	45 d after discharge to home	Government and/or university
61	Spain	community - Home care	Adults in a home care program with ADL difficulties and with a caregiver	65+	At risk (MNA 17-23.5)	I=72, C=101	Primary health care nurses (after receiving training) monitor at home every m	no nutrition intervention, visited at baseline, at 6 m and at 12 m	12 m	6 m and 12 m	Government and/or university

							for 6 m and at 12 m				
62	Japan	hospital	Patients with hip fracture	65+	Malnourished or at risk	Target n=60	ONS	none	NR	NR	Government and/or university
63	Denmark	community - after hospital discharge	Patients after hospital discharge living at home and alone	75+	Malnourished (MNA<24)	I1=73, I2=68, C=67	nutrition follow-up care at 1 w, 2 w and 4 w after discharge by clinical dietitian either at home in 45 min visit (I1) or by phone (I2)	none	4 w	30 d and 90 d	NR
64	China	community - after hospital discharge	Patients in polyclinic or inpatient ward prior to discharge	65-110	Malnourished or at risk (MUST>=1)	Enrolled n=811	ONS 1	ONS 2	180 d	180 d	(Co)funding by industry (and supplements provided by industry)
65	China	hospital	Community hospital patients	65-85	Malnourished or at risk (MNA-SF <12 and low muscle mass (<90% of normal level) and/or at least 1 type of micronutrient deficiency symptom in hidden hungry evaluation questionnaire)	Enrolled n=78	ONS powder	none	12 w	12 w	Government and/or university
66	Portugal	community	Outpatients with Alzheimer's' disease from a psychogeriatric department of a psychiatric hospital, living at home with their family and have an active caregiver	65+	Malnourished or at risk (weight loss >5% in previous y, MNA<24)	I=25, C=43	ONS and standard dietetic advice	standard dietetic advice	21 d	6 m	Government and/or university
67	Spain	hospital	Patients admitted to hospital for congestive heart failure,	65+	Malnourished or at risk (SGA B or C)	NR	High protein ONS	placebo ONS	NR	90 d, 180 d, 1 y, 2 y, 5 y and lifetime after discharge	(Co)funding by industry (and supplements

			acute myocardial infarction, pneumonia, COPD								provided by industry)
68	South Korea	community	(Pre)frail adults at welfare centres	70-85	Malnourished or at risk (MNA <=23.5)	I1=40, I2=40, C=40	protein powder (200 kcals, 9.3 g whey protein). Both a 1.2 (I1) and a 1.5 g protein / kg BW / d group (I2)	placebo powder (200 kcals, 0 g protein). 0.8 g protein / kg BW/d group	12 w	6 w and 12 w	Government and/or university
69	Denmark	community after discharge	Patients admitted to geriatric ward	65+	Malnourished or at risk (NRS-2002: BMI<20.5 and/or weight loss within last 3 m and/or reduced intake in the previous w and/or severely ill)	I=74, C=76	Individual dietary plan made by dietitian including advice on intake after discharge, based on food and combined with ONS when relevant. Home visits after discharge by nurse to manage dietary intake.	none	8 w after discharge	3 m after discharge	Government and/or university
70	Italy	community	patients from out-patient service for geriatric evaluation	80+	Malnourished (MNA<17)	I=78, C=77	Branched chain amino acids powder	brochure on consequence s of malnutrition and dietary recommenda tions	NR	1 m and 2 m	Government and/or university
71	USA	hospital	Hospitalized patients with chronic heart failure, acute MI, pneumonia or COPD	65+	Malnourished or at risk (SGA B or C)	I=14, C=16	ONS (350 kcal, 20 g protein)	ONS (48 kcal, 0 g protein)	90 d after discharge	90 d after discharge	(Co)funding by industry and industry employee is (co)author
72	Netherlan ds	nursing home	Dysphagia residents admitted to, or living in, a	65+	Malnourished or at risk (based on SNAQ^{RC})	Target n=156	standard treatment + pre-thickened ONS	standard nutritional + dysphagia management	12 w	12 w	(Co)funding by industry (and supplements

			somatic or psychogeriatric ward in nursing homes								provided by industry)
73	Taiwan	mixed setting	Patients with primary diagnosis pneumonia admitted to hospital	>65	Malnourished (BMI<18.5 or MNA-SF<=7)	I=39, C=43	Dietitian counselling involving family care givers, individualized nutritional plan, tracking after discharge by phone	standard nutritional supplements by hospital and no dietary advice	6 m	6 m	Government and/or university
74	Sweden	community - after hospital discharge	Patients admitted to participating wards with no or moderate cognitive impairment	65+	Malnourished or at risk (MNA score<23.5)	I1=168, I2=170, I3=169, C=164)	I1: dietary advice once just before discharge; I2: ONS after discharge; I3: both	none	6 m	between 3.4 and 8.2 years	Government and/or university
75	Japan	hospital	Acute stroke patients admitted to neurosurgical and neurology wards	>65	Malnourished (MUST>=2 or serum albumin<=3.0 g/dL)	I=64, C=64	individualized nutritional treatment, caloric requirement calculated by dietitian. Meals provided orally or enterally (tube feeding). Intervention until discharge hospital	standard diet (BW (kg) * 25 kcal)	until discharge	At discharge or 3 m after stroke onset	NR

ADL, activities of daily living; AMI, Acute myocardial infarction; BMI, body mass index; BW, body weight; CHF, Chronic heart failure; CPOD, Chronic obstructive pulmonary disease; d, days; DSM-IV, Diagnostic and Statistical Manual of Mental Disorders fourth edition; %en, percentage of energy; GP, general practitioner, m, months; MNA-SF, Mini Nutritional Assessment short form; MUAC, Mid-upper arm circumference; MUST, Malnutrition Universal Screening Tool; NR, not reported; NRS-2002, Nutrition Risk Screening 2002; ONS, Oral nutritional supplements; PNA, Pneumonia; SGA, Subjective Global Assessment; $SNAQ^{RC}$, Short Nutritional Assessment Questionnaire residential care; Ref, reference; TSF, Triceps skinfold; w, weeks.
