# Supplementary material for: Towards developing a Core Outcome Set for malnutrition intervention studies in older adults: a scoping review to identify frequently used research outcomes
Source: Eur Geriatr Med. 2022 Mar 12;13(4):867–79. doi: 10.1007/s41999-022-00617-5 (PMC9378339; doi:10.1007/s41999-022-00617-5)
Supplement: Supplementary file 3 — Supplementary file3 (DOCX 26 KB) [file 41999_2022_617_MOESM3_ESM.docx]

Table S3. Overview of the outcomes in the 60 randomised controlled trials included

| **Reference** | **Primary outcome(s)** | **Secondary outcome(s)** |
| --- | --- | --- |
| 13 | height, weight, dietary intake (energy and protein intake, 3 d counts), pressure ulcer, blood marker (blood count, WBC, total serum protein, serum albumin, serum cholesterol, serum transferrin, plasma zinc) |  |
| 14 | weight, skinfold (triceps), body circumference (MUAC) |  |
| 15 | Functional limitation (Mahoney) |  |
| 16 | dietary intake (energy and protein, 3 d dietary record), handgrip strength (Vital Sign TM), malnutrition status (MNA), weight, BMI |  |
| 17 | blood marker (albumin, pre-albumin, transferrin, IGF, CRP, PINI), weight, BMI, muscle mass (FFM and ASM, DXA), skinfold (triceps), body circumference (MUAC), handgrip strength |  |
| 18 | weight, dietary intake (energy intake, 4-d dietary record) |  |
| 19 | handgrip strength (Martin), muscle strength (maximum voluntary isometric strength of knee extensors and elbow flexors, Microfet2), functional performance  (TUG), quality of life (SF-36) | skinfold (triceps, sub-scapula, supra-iliac), body circumference (MUAC, calf) |
| 20 | weight (power calculation), height, malnutrition status (MNA), dietary intake (energy and protein intake, dietary record) |  |
| 21 | physical activity, dietary intake (4-d food diary), bone (BMD spine hip and whole body, DXA), muscle mass (DXA), quality of life (well-being, questionnaire), weight, height, BMI, body circumference (MUAC), blood marker (Hb, serum electrolytes, urea, creatinine, albumin, calcium, phosphate, serum 25-OHD, CTX, serum bone specific alkaline phosphatase and osteocalcin) |  |
| 22 | weight (power calculation), BMI | dietary intake (3-d dietary diary), handgrip strength, anthropometry, quality of life (EQ-5D), health care use, cost |
| 23 | weight (weight loss for power calculation), muscle mass (DXA), malnutrition status (MNA), blood marker (serum albumin, CRP), hospitalization, pressure ulcer, institutionalization, dietary intake (24-h recall), cognition (MMSE), functional limitation (Katz ADL index), eating behavior scale. |  |
| 24 | weight | body circumference (MUAC, MUAMC), skinfold (triceps), handgrip strength (Takei), height, dietary intake (energy intake, 24-h recalls) |
| 25 | dietary intake (energy intake, three 24-h recalls), body weight, malnutrition status (MNA), blood marker (fasting glucose, lipids, Hb, albumin, pre-albumin, lymphocyte count, ESR, ferritin, CRP, vitamin B12, folic acid), cognition (Pfeiffer), eating disorders (Blandford scale), depression (GDS), functional limitation (FAST). |  |
| 26 | functional limitation (SF-36) | nutritional measures, weight, muscle mass (lean body mass, DXA), anxiety and depression (hospital anxiety and depression scale), functional limitation (Barthel index), functional performance (TUG), handgrip strength |
| 27 | health care use, malnutrition status | dietary intake, blood marker |
| 28 | BMI, weight (power calculation), height, peak expiratory flow, blood marker (IGF-I and BPs, HDL, LDL, total cholesterol, triglycerides, 25-OHD, folate, zinc), functional limitation (Katz ADL index), handgrip strength (Harpenden), cognition (MMSE), quality of life (SF-36) |  |
| 29 | functional limitation (FIM) | length of stay, discharge disposition (home/not home), functional performance (2 and 6 min walk test) |
| 30 | dietary intake (energy and protein intake, food intake protocols), weight, height, BMI, body circumference (MUAC, calf), malnutrition status (MNA), muscle mass (FFM, BIA), handgrip strength (Mechatronic), peak expiratory flow, functional limitation (Barthel index), quality of life (SF-36) |  |
| 31 | hospitalization, quality of life (SF-36) | weight, anthropometry, handgrip strength (Stoelting Hean), dietary intake (24-h food recall), adverse events, frailty (Fried), functional performance (15 feet walk time), falls, malnutrition status (MNA), mortality, blood marker (testosterone, CRP, PSA, hematocrit, electrolytes, glucose, HDL, LDL, cholesterol, TG, hemoglobin, bilirubin) |
| 32 | functional limitation (Barthel index) | handgrip strength (Takei), functional performance (Sit to Stand test - 10 full stands), weight, BMI, quality of life (EQ-5D), physical activity (RT3 tri-axial tracker), dietary intake (3-d dietary record), adverse events, adherence |
| 33 | weight, muscle mass (FFM, total body water using deuterium dilation), resting energy expenditure (indirect calorimetry), nitrogen balance (urea analysis), dietary intake (energy intake, 3-d dietary record), blood marker (prognostic inflammatory and nutritional index) |  |
| 34,35 | weight (power calculation on percentage of patients becoming malnourished (>=5% weight loss)), height, muscle mass (ECW, ICW, fat and FFM, lean tissue mass, BCM was calculated, BIS), body circumference (MUAC), skinfold (triceps) | handgrip strength (Takei), quality of life (SF-36 and EQ-5D), self-perceived health (VAS), length of stay, dietary intake (energy and protein intake, registration forms |
| 36 | dietary intake (energy intake, three 24-h recalls) |  |
| 37 | weight, height, body circumference (MUAC), handgrip strength (JAMAR), functional limitation (Barthel index), functional performance (gait speed 2.44 m walk), complications, length of stay, rehospitalization, mortality |  |
| 38 | mortality (power calculation) | malnutrition status (MNA), cognition (MMSE), Charlson Comorbidity Index, dietary intake (24-h recall, FFQ), blood marker (albumin, cholesterol, Hb, total lymphocyte count, transferrin), depression (GDS), functional limitation (Barthel index) |
| 39 | functional limitation (self-reported disability score), functional performance (SPPB) | dietary intake, weight, body circumference (MUAC), handgrip strength, functional performance (TUG, usual gait speed, one leg stands), blood marker (BUN, creatinine) |
| 40 | malnutrition status (MNA) | height, weight, body circumference (calf), dietary intake (FFQ), blood marker ((pre)albumin, transferrin, hemoglobin, hematocrit, lymphocyte count, serum iron), age, sex, medical history, functional limitations (Barthel index), cognition (Pfeiffer's test), depression (Yesavage Depression Scale), knowledge acquisition, social risk (family socio-scale) |
| 41 | weight change, mortality, number of presentations to emergency, rehospitalization, length of stay (power calculation), cost of hospital admission |  |
| 42 | rehospitalization | mortality (hospital database), handgrip strength (JAMAR), functional performance (30 sec chair stand), functional limitation (Morton Mobility Index, Mob T scale, functional Recovery Scale), weight, BMI, dietary intake (energy and protein intake, 4-d dietary record or 24-h recall), health care use (use of home care, meal on wheels, day care with exercise) |
| 43 | dietary intake (energy intake, 3-d weighed intake diaries) | weight, BMI, body circumference (MUAC) |
| 44 | functional limitation (disability by physical functioning, PF), functional performance (SSPB) | functional performance (TUG), handgrip strength (Tanita), dietary intake (3 24-h recalls), weight, body circumference (MUAC) |
| 45 | dietary intake (energy intake, three 24-h recalls), weight |  |
| 46,47 | weight, functional performance (SPPB), handgrip strength (JAMAR) | dietary intake (energy and protein intake, food diary or 24-h recall), muscle mass (FFM, BIA), cost, quality of life (EQ-5D) |
| 48 | dietary intake (dietary record), weight, height, BMI, body circumference (MUAC, calf), malnutrition status (MNA-SF), cognition (MMSE), depression (GDS), functional limitation (Mahoney and Barthel index), handgrip strength (Vigorimeter Martin), functional performance (gait speed 4 m usual walking speed), quality of life (QUALIDEM) |  |
| 49 | mortality | chemo management (dosage, changes and arrest), grade 3-4 toxicities including severe infections, weight, prescription enteral or parenteral nutrition, hospitalization |
| 50 | weight, BMI, dietary intake (energy and protein intake, 4-d dietary record), handgrip strength (JAMAR), functional performance (30 sec chair stand), functional limitation (Morton Mobility index, Barthel index), quality of life (EQ-5D-3L), health care use (use of social services; (re-)hospitalization (hospital patient register), mortality (hospital patient register) |  |
| 51 | weight, height, BMI, body circumference (MUAC, calf), malnutrition status (MNA-SF) |  |
| 52,53 | weight | appetite, dietary intake (% of serving consumed), pressure ulcer, diarrhea, falls, infection episodes, antibiotic days, mortality, cost |
| 54 | weight, BMI, acceptance intervention by patients, retention rate, feasibility, malnutrition status (MUST), body circumference (MUAC), skinfold (triceps), handgrip strength (Smedley), dietary intake (daily food charts), health care use (ICER), health state (EQ5D-5L), appetite and dietary satisfaction (VAS), quality of life (COOP) |  |
| 55 | weight, malnutrition status (MNA-SF), total number diagnoses, number of prescribed drugs, dietary intake (energy intake 3-d fluid and food record, power calculation), blood marker (albumin, transthyretin, IGF-I, calcium, zinc, 25-OHD, CRP, Apo A, Apo B, total cholesterol, LDL cholesterol, triglycerides, coagulation factors), appetite (VAS), functional performance (SPPB), handgrip strength (Martin), peak expiratory flow (Vitalograph) |  |
| 56 | composite event: mortality or non-elective rehospitalization | rehospitalization, mortality, length of stay, functional limitation (Katz ADL index), malnutrition status (SGA), blood marker (25-OHD) |
| 57 | muscle mass (lean body mass) | weight |
| 58 | functional limitation (Barthel index, power calculation) | functional performance (30-sec chair stands), handgrip strength, functional limitation (Cumulated Ambulation Score, Mob-T), quality of life (SF-36), depression (Depression List, GDS), malnutrition status (MNA) |
| 59 | BMI, malnutrition status (MNA), handgrip strength (JAMAR), functional limitation (Katz ADL index, Lawton index), dietary intake (weighed 3-d records), blood marker (vitamin B1,2,6,9,12 and 25-OHD, homocysteine, selenium, albumin, prealbumin, CRP) |  |
| 60 | (re)hospitalization | dietary intake (3 24-h recalls), program satisfaction |
| 61 | malnutrition status (MNA, power calculation) | height, weight, BMI, body circumference (MUAC), dietary intake (FFQ), blood marker (albumin and pre-albumin, Hb, hematocrit, cholesterol), functional limitation (Barthel index), cognition (Pfeiffers test), depression (GDS). |
| 62 | rehabilitation | handgrip strength, body circumference (calf), functional performance (walking speed), malnutrition status, cognition (FAST) |
| 63 | rehospitalization |  |
| 64 | weight, rehospitalization | muscle mass (FFM, BIA), length of stay, hospitalization |
| 65 | malnutrition status (MNA-SF) | muscle mass (skeletal muscle mass, BIA), BMI, body circumference (calf), handgrip strength, functional performance (6 m gait speed), hunger (Hidden Hunger Assessment Scale), dietary intake, malnutrition status (DETERMINE) score, health lifestyle questionnaire, self-satisfaction questionnaire |
| 66 | weight, BMI, skinfold (triceps), body circumference (MUAMC), muscle mass (FFM, BIA), malnutrition status (MNA), handgrip strength (Smedley), functional limitation (Barthel index, Lawton index), cognition (MMSE) |  |
| 67 | cost |  |
| 68 | muscle mass (ASM/ht2, ASM/BMI and ASM/fat ratio, DXA, power calculation) | frailty (modified CHS), handgrip strength (adjusted for sex and BMI, Takei), functional performance (SPPB TUG), functional limitation (Korea Instrumental), cognition (MMSE), malnutrition status (MNA), weight, height, body circumference (MUAC, calf), blood marker (CRP, C-peptide, ILGF-I, blood cell count) |
| 69 | weight (power calculation) | handgrip strength (SAEHAN), functional limitations (Barthel index), rehospitalization, mortality |
| 70 | body circumference (MUAC, calf), handgrip strength (MSD), functional performance (TUG, 30 s sit to stand test, 4m gait speed), risk of falls (Tinetti Gait and Balance Instrument), mitochondrial ATP production | cognition (MMSE), dietary intake (recall on 7 d), BMI, skinfold (triceps, sub-scapula, abdominal), self-perceived health, blood marker (mitochondrial biogenesis and fusion, activity of peripheral blood mononuclear cells, oxidative stress (plasma thioarbituric acid reactive substances)), functional limitation (Katz ADL index), depression (GDS), comorbidity (Cumulative Index Rating Scale) |
| 71 | dietary intake (24-h recalls) |  |
| 72 | weight | dietary intake, quality of life (QoL), functional limitation (activities of daily living), blood marker (micronutrient status), body circumference (MUAC, calf), dysphagia severity, hydration status, complications |
| 73 | malnutrition status (MNA-SF) | weight, height, skinfold (triceps), body circumference (MUAC), calorie needs (based on IBW and PA), dietary intake (24-h recall), blood marker (albumin, WBC, lymphocyte, complete blood count), length of stay, rehospitalization |
| 74 | mortality | quality of life, health care use, functional limitation |
| 75 | functional limitation (FIM, power calculation) | cognition (FIM), weight, body circumference (MUAC, thigh, calf) |

ADL, activities of daily living; ASM, appendicular skeletal mass; BIA, Bioelectrical impedance analysis; BMD, bone mass density; BMI, body mass index; BUN, Blood urea nitrogen; BW, body weight; CRP, c-reactive protein; CTX, C-terminal telopeptide; DXA, bone density scan; ECW, extracellular water; FAST, Functional assessment screening tool; FFM, fat-free mass; FFQ, Food frequency questionnaire; FIM, Functional independence measure; GDS, Geriatric depression scale; GP, general practitioner; HDL, High-density lipoprotein; IBW, ideal body weight; ICW, intracellular water; IGF, Insulin-like growth factor; LDL, Low-density lipoprotein; MMSE, Mini-Mental State Examination; MNA, Mini Nutritional Assessment; MNA-SF, Mini Nutritional Assessment short form; MUAC, Mid-upper arm circumference; MUST, Malnutrition Universal Screening Tool; PA, physical activity; PSA, prostate-specific antigen; SF-36, Short form health survey; SGA, Subjective Global Assessment; SPPB, Short Physical Performance Battery; TG, triglycerides; TSF, Triceps skinfold; TUG, Timed up and go; VAS, Visual analogue scale; WBC, white blood cell count.
