# Supplementary material for: Towards developing a Core Outcome Set for malnutrition intervention studies in older adults: a scoping review to identify frequently used research outcomes
Source: Eur Geriatr Med. 2022 Mar 12;13(4):867–79. doi: 10.1007/s41999-022-00617-5 (PMC9378339; doi:10.1007/s41999-022-00617-5)
Supplement: Supplementary file 4 — Supplementary file4 (PDF 81 KB) [file 41999_2022_617_MOESM4_ESM.pdf]

Table S4. Overview of all used outcomes and outcome domains as well as primary outcomes and outcome domains in the 60 included randomized controlled trials and their frequency* of use.

Variable	All RCTs (n=60)		Setting							
			Community (n=26)		Hospital (n=13)		Long- term care (n=13)		Other (n=8)	
	outcome	primary outcome	outcome	primary outcome	outcome	primary outcome	outcome	primary outcome	outcome	primary outcome
Body weight / BMI	45	30	19	10	9	7	13	12	4	1
Dietary intake	37	18	17	5	5	3	12	10	3	
Functional limitation	29	17	15	7	3	3	5	4	6	3
Handgrip strength	28	14	15	5	5	2	6	6	2	1
Body circumference	24	11	10	3	7	4	6	4	1	
Malnutrition status	23	16	7	4	4	2	8	8	4	2
Functional performance	20	9	13	6	4	1	2	2	1	
Blood marker	19	9	8	2	2	2	5	4	4	1
Quality of life	15	8	9	4	1		4	3	1	1
(Re)hospitalization	15	9	9	6	2	2			4	1
Muscle mass	13	9	6	4	4	3	1	1	2	1
Cognition	12	5	6	2	2		2	2	2	1
Mortality	12	7	6	3	2	2	1		3	2
Skinfold	9	5	4	1	3	3	1	1	1	
Depression	8	2	4				2	2	2	
Length of stay	7	2	1		4	2			2	
Care use	6	3	4	1			1	1	1	1
Cost	5	2	2		2	2	1			
Fall	4	1	3	1			1			

Pressure ulcer	3	2	1	1			2	1		
Peak expiratory flow	3	3					2	2	1	1
Appetite	3	2					3	2		
Complication	2	1			1	1	1			
Frailty	2		2							
Physical activity	2	1	2	1						
Self-perceived health	2			1		1				
Acceptance intervention	1	1					1	1		
Adherence to intervention	1		1							
Adverse events	1		1							
Antibiotic days	1				1					
Anxiety	1								1	
Bone mineral density	1	1	1	1						
Calorie need	1								1	
Chemo management	1		1							
Comorbidity	1		1							
Diarrhea	1						1			
Dietary satisfaction	1	1					1	1		
Discharge disposition	1				1					
Dysphagia severity	1						1			
Eating behaviour	1	1	1	1						
Eating disorder	1	1					1	1		
Feasibility intervention	1	1					1	1		
Grade 3-4 toxicities including severe infection	1		1							
Health lifestyle	1				1					
Health state	1	1			1	1				

Hunger	1				1					
Hydration status	1						1			
Infection episodes	1						1			
Institutionalization	1	1	1	1						
Knowledge acquisition	1		1							
Mitochondrial ATP production	1	1	1	1						
Nitrogen balance	1	1			1	1				
Number of prescribed drugs	1	1					1	1		
Number of presentations to emergency	1	1			1	1				
Prescription enteral of parental nutrition	1		1							
Program satisfaction	1		1							
Rehabilitation	1	1			1	1				
Registration forms	1				1					
Resting energy expenditure	1	1			1	1				
Retention rate	1	1					1	1		
Self-satisfaction	1				1					
Total number diagnoses	1	1					1	1		

* an empty cell indicates frequency 0.
